# Factors Underpinning the Shift to Eveningness during Early Adolescence: Pubertal Development and Family Conflicts

**DOI:** 10.1007/s10964-022-01708-z

**Published:** 2022-11-26

**Authors:** Juan F. Díaz Morales, Cristina Escribano, Yaiza Puig-Navarro, Konrad S. Jankowski

**Affiliations:** 1 Individual Differences, Work and Social Psychology, Faculty of Psychology, Complutense University of Madrid, Campus de Somosaguas, s/n, 28223 Pozuelo de Alarcón, Madrid Spain; 2 Cardenal Cisneros University College, University of Alcalá, Alcala de Henares (Madrid), Spain; 3 Faculty of Psychology, University of Warsaw, Warsaw, Poland

**Keywords:** Chronotype, Sleep, Eveningness, Adolescence, Puberty

## Abstract

Biological and psychosocial factors have been related to the shift to eveningness during early adolescence but it is necessary to study them from a longitudinal perspective. This longitudinal study examined the contribution of these factors to the onset of a shift towards eveningness in early adolescence. A sample of 440 (49.9% boys) Spanish adolescents were assessed for pubertal development, family conflicts, and morningness/eveningness. The same measures were taken twice at the age of 12 and one year later (T1: *M* = 12.47, *SD* = 0.75 and T2: *M* = 13.64, *SD* = 0.78). Pubertal development and family conflicts were considered predictors of morningness/eveningness in a mixed-effects multilevel model. The developmental shift towards eveningness appeared in girls but not in boys. The shift was related to more advanced pubertal development and more conflicts in the family. This study has implications for shaping healthy sleep habits in adolescents and possible interventions focused on family dynamics.

## Introduction

During adolescence there is a progressive change towards greater eveningness, a delay in the phase of circadian rhythms which is manifested in the delay in going to bed and getting up, as well as in the preference for activities in the afternoon-evening. This change is due to both biological (i.e., hormonal changes that occur around the onset of puberty) and social factors (e.g., search for autonomy and refusal to follow social and family norms, peers influence, or mobile use at night). Previous research that has studied the relationship between eveningness-biological-psychosocial variables have used cross-sectional designs, finding that both pubertal development and family conflicts predicted eveningness (Díaz-Morales et al., 2014). In order to study more in depth this complex interrelation, the aim of this study was to test this interrelation adopting a longitudinal approach, considering also sex differences, because previous studies have found a greater tendency to eveningness in girls in early adolescence, which could be related to an earlier pubertal development.

### The Shift to Eveningness during Adolescence: Biological Factors

Individuals have different preferred schedules for their sleep and daily activities that are reflected on the morningness/eveningness continuum. Morningness/eveningness reflects individual differences both in the behavioural patterns and circadian rhythms, indicating a trend towards an advanced or delayed phase (Kerkhof, 1985). Based on the morningness/eveningness continuum, people can be further assigned to a circadian typology (morning, intermediate and evening types). A person with a morning orientation prefers morning activities, gets up easily in the early morning, and is more alert in the morning than in the evening, whereas people with an evening orientation prefer evening activities, and their level of alertness increases as the day progresses (Adan et al., 2012).

Morningness/eveningness is not constant but rather changes throughout the lifespan. Cross-sectional research demonstrates that individuals display low eveningness during childhood, a shift towards eveningness during adolescence, and a slow decrease in eveningness starting in the late teens and early 20’s through adulthood (Fischer et al., 2017).

The shift to eveningness during adolescence occurs around the onset of puberty. Pubertal development was related to morningness/eveningness, with adolescents higher on eveningness being more mature (Carskadon & Acebo, 1993; Laberge et al., 2001; Randler et al., 2009). Hagenauer et al. (2009) concluded that biological factors can account for the shift towards eveningness during adolescence given that (1) bedtime delay was observed earlier in girls than in boys coinciding with their earlier pubertal development, (2) the shift towards eveningness has been observed in many different countries (but see West Africa, Borchers & Randler, 2012), and (3) adolescents continue to show a delayed circadian phase even in laboratory studies where social influence is limited. At the same time, their circadian timing system becomes delayed and drives them to wake up later, and previous research proposed the influence of gonadal hormones on sleep patterns (Jenni et al., 2005) – hypothesis waiting for verification in adolescents, with premises found already in adults (Jankowski et al., 2019). Other researchers have shown that girls sleep later as menarche approaches (Beal et al., 2016), and that the first manifestations of puberty occur earlier in girls (Muñoz Calvo & Pozo Román, 2011; Susman et al., 2010). Also, studies found differences in rise times on weekdays and sleep length on weekends, with girls waking up later and sleeping longer on weekends (Collado et al., 2012). These delayed rise times among girls during weekends, without restrictions from school schedules, might be interpreted as a result of an earlier onset of puberty (Randler, 2011). Given that the timing of puberty differs according to sex and most studies used the cross-sectional design, longitudinal studies are needed to analyse the differences according to sex in the shift to eveningness.

### The Role of Psychosocial Factors

Maturational changes have an influence on the shift towards eveningness during adolescence but also on psychosocial functioning (Hagenauer & Lee, 2012; Steinberg, 2002). Along with biological factors, a variety of psychosocial factors influence adolescents’ sleep, and their potential reciprocal interactions are thought to be complex. The possible influencing factors have been clustered into physiological (e.g., circadian patterns of melatonin secretion), psychiatric (e.g., affective disorders), sociocultural and psychological factors (Brand et al., 2009). As adolescents get older, both their academic responsibilities and interest in night life increase, and they feel independent from parental supervision (Becker et al., 2015). All these psychosocial factors may contribute to the well-known shift towards eveningness during early adolescence, although studies that have evaluated both biological and psychosocial factors jointly are scarce (Díaz-Morales et al., 2014). Joint consideration of various factors is especially important because the relationship between the delayed sleep-wake rhythm during adolescence and psychological functioning has been shown to be bidirectional (Bajoghli et al., 2013) or determined not only by biology. For example, according to Takeuchi et al. (2001), several factors, such as environmental (light and temperature), social (family life and school schedules), physiological (meal timing), and psychological (parental discipline about sleep habits) factors, may act as *zeitgebers* of the sleep-wake rhythm in adolescents.

One important psychosocial factor related to sleep among adolescents is family functioning (Bajoghli et al., 2013) and family rules and routines (Buxton et al., 2015). Khor et al. (2021) performed a meta-analysis and review of up to 12 domains of parental factors that may be related to the quantity and quality of sleep in adolescents. Family conflicts were of sound relevance for poor sleep in adolescents. Concordance between adolescents’ and their parents’ sleep and well-being has also been found (Kalak et al., 2012). It seems that a positive home atmosphere favours high sleep quality among offspring (Sasser et al., 2021), and total sleep time increases with more strict parental rules, such as earlier bedtimes (Adam et al., 2007). Establishing rules of sleep hygiene, such as by limiting caffeine intake and maintaining a regular bedtime and keeping the use of electronic and media devices out of the bedroom, are related to better age-appropriate levels of sleep (Buxton et al., 2015). The overall family climate and whether adolescents perceive their home environment as safe and predictable can also influence their sleep outcomes (Meltzer et al., 2021).

On the other hand, parents and adolescents have divergent views on a host of domains of family functioning (Van Petegem et al., 2012). Prior studies have indicated that parents and adolescents largely agree that daily life topics (e.g., household chores/doing laundry and completing homework) are the issues around which conflict interactions typically transpire (De Los Reyes et al., 2013). Adolescents who live in families with high levels of conflicts, parental psychopathology, and various forms of family risks tend to have worse sleep (El‐Sheikh & Kelly, 2017). Adolescents exposed to family conflicts likely experience a lack of safety and increased emotional arousal, which negatively impacts their sleep quality, leading to greater daytime sleepiness (Roblyer & Grzywacz, 2015). The sensitization hypothesis proposes that repeated exposure to high levels of family conflict is associated with greater child emotional and behavioural reactivity in response to conflicts and, more broadly, with an array of psychological problems (Cummings & Davies, 2002). Given that adolescents tend towards higher eveningness and parents tend towards lower eveningness, both parties would be out of sync in the assessment of rules that govern family life affecting daily routines, as demonstrated in young children (Zimmermann, 2016). Collectively, the literature suggests reciprocal relations between family functioning and sleep, and supports integrated biopsychosocial and contextual framework proposed by Becker et al. (2015) to the study of sleep in adolescence, considering sleep to be interwoven with the biological, psychosocial, and contextual factors of adolescents’ lives—not only at any given time point, but also across development.

### From Cross-Sectional to Longitudinal Design

Considering studies analysing joint effects of biological and psychosocial factors on the shift towards eveningness during the age of puberty, Randler et al. (2009) found that higher pubertal development was associated with a higher eveningness and that adolescents without parental monitoring went to bed later and were evening types. Díaz-Morales et al. (2014) showed that advancement in puberty was accompanied by an increase in functional autonomy and family conflicts that together were related to the increase in eveningness. Age (from 12 to 16) was related to more advanced pubertal status, and these two developmental indices were linked to greater functional autonomy, more conflict frequency and greater eveningness. The mentioned study, however, did not analyse puberty in a longitudinal manner and did not focus on potential sex differences, which seems especially important considering that pubertal development in girls occurs earlier.

## Current Study

The shift to eveningness during adolescence is influenced by both biological and psychosocial factors, and therefore it is necessary to analyse them both together with a longitudinal methodology, while considering possible differences between boys and girls. Consequently, the aim of this prospective study was to analyse the role of pubertal development and conflicts with parents in the shift towards eveningness during adolescence with a focus on sex differences. It is hypothesized that pubertal development and family conflicts are associated with eveningness, with the association between pubertal development and eveningness being stronger in girls.

## Methods

### Participants

A total of 495 high school students were recruited to participate in a 1-year longitudinal study. Of this total, 440 adolescents (223 girls and 217 boys) completed the measures twice and were considered in the analyses. The first measurement was taken in the first year of secondary education in the Spanish education system (8th grade according to the UK education system or Grade 7 in the US education system) (*Mean age* = 12.47, *SD* = 0.75). The second measurement was taken a year later in the second year of secondary education (*Mean age* = 13.64, *SD* = 0.78). All participants were studying in four public high schools in an urban area located in the east of the Community of Madrid (Spain), which comprises several cities with a population of over 100,000 people each. Approximately 35% of the working population is hired in the industrial sector. Considering the level of education and the income level of the parents, the most represented socioeconomic status in this study was middle class. The board of directors at schools authorized the study after obtaining parents’ permission.

### Measures

#### Morningness/Eveningness

The Morningness/Eveningness Scale for Children (MESC; Carskadon & Acebo, 1993) assesses chronotype with items about the preferred timing of various activities and performance at various times of day. Children were posed a scenario, and they had to identify the statement that best fit them (e.g., “Gym class is set for 7:00 in the morning, how do you think you will do? Answer choices: “My best!”, “Okay”, “Worse than usual”, and “Awful!”). Responses are scored on a 1–4 or 5 scale and are summed to the total score ranging from 10 to 43, with lower scores indicating a preference for eveningness. The scale has been adapted to different cultural contexts, and validity has been verified with sleep habits, self-assessments of the level of alertness, physical and academic performance, and mood (Díaz-Morales, 2015). In the present study, internal consistency was *α* = 0.72.

#### Pubertal development

The Pubertal Development Scale (PDS; Carskadon & Acebo, 1993) is designed to represent the Tanner staging categories and assesses pubertal development by self-report. Carskadon & Acebo (1993) tested the validity and reliability of their scale and recommended it for use in noninvasive settings and large-scale survey studies. The scale includes three questions on growth in height, body hair changes, and skin changes such as pimple formation for both genders; two items on voice deepening and facial hair growth concerning boys only; and three questions on breast growth, menstruation, and age at menarche concerning girls only. These items were rated on a 4-point scale ranging from 1 (*not yet started*) to 4 (*seems complete*). The menstruation item, which was rated as “yes” or “not”, was coded as 4 if the answer was yes or 1 if the answer was not. The total score was the average of all items. The percentage of non-response in any of the items in the scale ranged from 0.89–3.58% in girls and 2.3–6.4% in boys. The Cronbach’s alphas were 0.62 (girls) and 0.76 (boys). The internal consistency was similar to that obtained by Carskadon & Acebo (1993; *α* = 0.67–0.70) and Díaz-Morales et al. (2014; *α* = 0.61–0.75).

#### Family conflict

The Family Conflict Scale (Parra & Oliva, 2002) is composed of a general question regarding 14 specific domains requesting declaration of frequency of conflicts in each domain. The general question was “We would like you to indicate if over the last month you have had any conflict with your parents about the following topics and who made the final decision about them”. The 14 items were related to (1) the time of returning home, (2) free time use, (3) time spent studying and for goal achievement, (4) peer groups socialized with during free time, (5) affairs, (6) sexual behaviour, (7) clothing style, (8) housework (cleaning and bedroom maintenance), (9) alcohol consumption and smoking, (10) drug consumption, (11) places gone with friends during free time, (12) money spending habits, (13) religion or political topics, and (14) goals and career aspirations. For each item, adolescents reported the frequency of conflicts (1 = no conflict, 2 = only a few conflicts, 3 = quite a few conflicts, and 4 = a lot of conflicts). In order to obtain a total score, scores for all conflict domains were summed. In the present study, internal consistency (Cronbach’s alpha) was *α* = 0.78, which is similar to that obtained by Díaz-Morales et al. (2014; *α* = 0.79).

### Data Analysis

The preliminary analysis involved a series of 2-way repeated-measures ANOVAs with the time of measurement set as a within-subjects factor (T1 = Time 1 and T2 = Time 2); Sex as a between-subjects factor (Girls, Boys) and Pubertal Development (PD), Family Conflicts (FC), and morningness/eveningness set as dependent variables.

Mixed-effects analysis was used as the main statistical tool, as it is particularly suited for longitudinal data because it takes into account the association between data points recorded from the same person over time. Since there are two moments of measurement, multilevel modelling was used to estimate within-subjects associations of morningness/eveningness with PD and FC as moderators. The Latent Change Score (LCS) was also considered but was discarded for the following reasons: in the first place, LCS allows bivariate analysis (for example, to what extent the change in morningness/eveningness is due to PD) but does not allow analysis of third variables, unless the group of participants was divided by the variable of interest (Sex or FC in the present study). Second, depending on the treatment of time in the analysis, two different types of longitudinal models can be distinguished: static and dynamic models. In static models, time is treated as a predictor in the model equation (growth curve models or multilevel/mixed-effects models), while in dynamic models (latent change score models), time is implicitly considered by the order of measurement occasions but is not explicitly used as a predictor, which is a limitation when attempting to characterize longitudinal changes as a function of a developmental process such as age or biological maturation (Estrada et al., 2020; Voelkle & Oud, 2015). Finally, in one two-times LCS model only five degrees of freedom are available in the data, although there are six parameters to be estimated. Therefore, the model is not identified (Estrada et al., 2018). For these reasons, a mixed multilevel analysis was conducted to account for repeated measures (T1 and T2) of morningness/eveningness (Table 1). Time (within-individual effect) was considered a predictor of morningness/eveningness. To test the moderation hypothesis, interactions of Time with FC and PD were added. FC and PD were grand-mean centred and were introduced into the model as fixed variables. Also, the intraclass correlation coefficient (ICC) was computed to examine the within-person variability of morningness/eveningness across time. Analyses were run using SPSS version 25 software (IBM Corporation, Armonk, NY).

**Table 1:** Multilevel analysis for morningness/eveningness predicted by family conflicts (FC), Pubertal Development Scale (PDS), Age, and Time (T1 vs. T2)

Parameter	Estimate	Std. Error	t	Sig.	95% Confidence Interval	
					Lower Bound	Upper Bound
Intercept	31.34	2.88	10.90	0.000	25.69	36.99
Time	0.74	0.44	1.69	0.091	−0.12	1.60
Sex (0 = girls)	0.57	0.50	1.15	0.250	−0.40	1.54
PDS	−0.59	0.37	−1.59	0.114	−1.31	0.14
FC	−0.12	0.03	−3.42	0.001	−0.18	−0.05
Time*Sex	−1.26	0.50	−2.52	0.012	−2.25	−0.28
Time*PDS	0.88	0.48	1.83	0.068	−0.07	1.83
Time*FC	−0.05	0.04	−1.19	0.234	−0.13	0.03
Age	−0.46	0.22	−2.03	0.043	−0.90	−0.01

*n* = 440; (0 = girls)

## Results

### Preliminary Analysis

Three repeated-measures ANOVAs for morningness/eveningness, FC and PD were performed while testing the intrasubject effect of Time, the intersubject effect of Sex and the interaction between both.

**Fig. 1:**
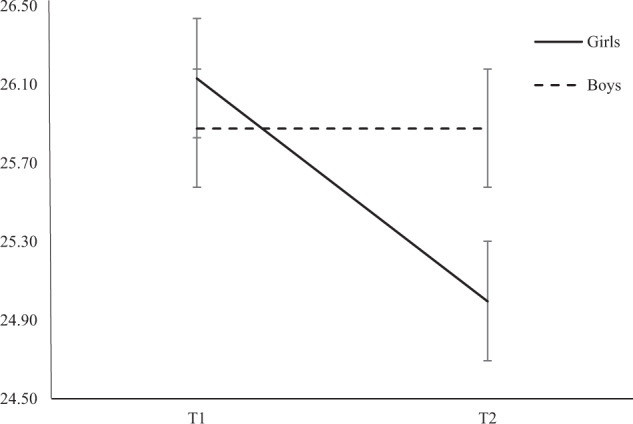
Interaction between time and sex on morningness/eveningness

The intrasubject effect of Time on morningness/eveningness was significant (F_438_ = 13.05, *p* < 0.001), indicating that morningness/eveningness decreased from T1 to T2. The intersubject effect of Sex was not significant (F_438_ = 0.12, *p* = 0.72), whereas the interaction of Time*Sex was significant (F_438_ = 3.45, *p* < 0.05), indicating that the shift towards eveningness was more pronounced in girls than in boys (Fig. 1).

The intrasubject effect of Time on FC was significant (F_438_ = 9.69, *p* < 0.01), indicating that FC increased from T1 to T2; the intersubject effect of Sex was not significant (F_438_ = 0.01, *p* = 0.93); and the interaction of Time*Sex was not significant (F_438_ = 0.12, *p* = 0.725).

Finally, the intrasubject effect of Time on PD was significant (F_438_ = 173.93, *p* < 0.001), indicating that PD increased from T1 to T2; the intersubject effect of Sex was significant (F_438_ = 9.28, *p* < 0.01), indicating higher PD scores in girls; and the interaction of Time*Sex was also significant (F_438_ = 9.28, *p* < 0.01), indicating that PD increased to a greater extent in girls than in boys.

The correlations between T1 and T2 for morningness/eveningness, FC and PD were positive and strong (Table 2).

**Table 2:** Means (*M*), standard deviations (*SD*) and Pearson correlations between Morningness/Eveningness (M/E), Family Conflicts (FC), Pubertal Development Scale (PDS), age and sex at Time 1 and Time 2

		Time 1				Time 2			
	*M* (*SD*)	M/E	FC	PDS	Age	M/E	FC	PDS	Age
Time 1									
M/E	26.00 (4.49)	–							
FC	19.90 (5.81)	−0.21**	–						
PDS	2.55 (0.62)	−0.09	0.13**	–					
Age	12.47 (0.75)	−0.18**	0.15**	0.17**	–				
Time 2									
M/E	25.25 (4.49)	0.53**	−0.16**	−0.06	−0.07	–			
FC	20.78 (5.92)	−0.15**	0.45**	0.03	0.10*	−0.25**	–		
PDS	2.87 (0.51)	−0.02	0.02	0.48**	0.09	−0.02	0.03	–	
Age	13.60 (0.78)	−0.17**	0.17**	0.18**	0.89**	−0.06	0.12*	0.12*	–
Sex	–	0.03	−0.01	−0.47***	0.12**	0.06	−0.01	−0.38**	0.08

Sex: 0 = girls; 1 = boys; **p < 0.01; ***p < 0.001, *n* = 440

### Mixed-Effects Analysis

The intraclass correlation coefficient (ICC = 0.519) shows that 51.9% of the variability in morningness/eveningness scores can be attributed to differences between adolescents, while the remaining 48.1% is due to evolution over time.

A multilevel analysis of morningness/eveningness revealed no significant differences according to Time and Sex, whereas FC and Time*Sex interactions were statistically significant (Table 1). Given that the Time*Sex interaction was statistically significant, the multilevel analysis was conducted for girls and boys separately (Table 3).

**Table 3:** Multilevel analysis for morningness/eveningness predicted by Family Conflicts (FC), Pubertal Development Scale (PDS), Age, and Time (T1 vs. T2) in Girls and Boys

Sex		Estimate	Std. Error	*t*	Sig.	95% Confidence Interval	
						Lower Bound	Upper Bound
Girls	Intercept	29.86	3.96	7.54	0.000	22.07	37.65
	Time	−0.66	0.52	−1.28	0.203	−1.67	0.36
	PDS	−1.02	0.51	−2.10	0.036	−2.01	−0.02
	FC	−0.14	0.05	−2.85	0.005	−0.23	−0.04
	Time*FC	−0.08	0.05	−1.46	0.146	−0.19	0.03
	Time*PDS	1.28	0.74	1.73	0.084	−0.17	2.73
	Age	−0.29	0.32	−0.90	0.370	−0.92	0.34
Boys	Intercept	33.32	4.06	8.20	0.000	25.33	41.31
	Time	0.66	0.55	1.20	0.229	−0.42	1.75
	PDS	−0.27	0.54	−0.50	0.616	−1.33	0.79
	FC	−0.09	0.05	−2.08	0.032	−0.19	0.00
	Time*FC	−0.02	0.06	−0.29	0.772	−0.13	0.10
	Time*PDS	0.61	0.65	0.95	0.342	−0.66	1.89
	Age	−0.60	0.32	−1.89	0.060	−1.23	0.03

*n* = 440; 223 girls and 217 boys

Multilevel analysis results show that the shift to eveningness over time was significant in girls but not in boys. An increase in FC was related to eveningness in both sexes, whereas greater PD predicted more eveningness only in girls. None of the interaction were significant.

## Discussion

Among factors related to the shift to eveningness during adolescence, pubertal development and family conflict have been considered in cross-sectional studies. Nevertheless, there was a gap in previous research as they did not consider both factors from longitudinal and gender perspectives. Using a longitudinal design, this study tested factors predicting the shift to eveningness in early adolescence with a particular emphasis on sex differences. The relevance of biological (pubertal development) and psychological (family conflict) variables were considered in examining this shift. The results indicate that progression from 12 to 13 years of age is accompanied by a shift to eveningness only in girls and that this shift is fuelled by an advance in pubertal development and increasing family conflict. Below, these results are discussed in more detail.

The sharp shift in eveningness during adolescence has been largely ascribed to biological and psychological factors occurring during this developmental stage (Roenneberg et al., 2004), although studies considering sex differences are scarce. The results of this study showing that the shift towards eveningness at the age of 13 appears in girls, but not in boys, are in line with the findings of Randler (2011), who analysed German adolescents aged 12–23 and concluded that the shift towards eveningness occurs earlier in girls. Nevertheless, present results show that pubertal development started in both sexes, although it was less advanced in boys. This suggests that there might be a certain level of pubertal development that switches in the shift towards eveningness, which in this sample occurred in girls and not in boys. Indeed, pubertal development occurred earlier and was progressing more rapidly in girls than in boys, as indicated by the higher PD scores in girls and their steeper increase over the study period. Consistent with these results, recent research shows that the timing of pubertal development (more advanced in girls) and hormone levels could explain changes in sleep habits primarily in girls (Foley et al., 2018). A similar outcome regarding pubertal development and eveningness was observed by Carskadon & Acebo, (1993) in their cross-sectional study of 11- to 12-year-old girls and boys, indicating that more pubertally advanced girls display more eveningness. The association between pubertal development and eveningness observed in the present results in girls supports the idea that pubertal changes influence the timing of sleep patterns in early adolescents, which is inferred from the results of previous cross-sectional studies (Beal et al., 2016; Díaz-Morales et al., 2014; Randler et al., 2009). Taken together, present and past studies indicate that pubertal development in girls is key to understanding their shift to eveningness in early adolescence. This finding is relevant considering the role of sleep in mental health during adolescence. Girls, whose sleep duration is shorter during early and middle adolescence, report more sleep problems and have more emotional symptoms than boys throughout adolescence (Kortesoja et al., 2020). Nevertheless, the need for further research considering sex differences needs to be highlighted since another longitudinal study testing the effects of eveningness in 14- to 19-year-olds found that eveningness increases risky behaviours and substance use in boys but not in girls, while a relationship with depressive symptoms was not found (Karan et al., 2021).

Interestingly, the role of family conflicts in shaping chronotype in early adolescence was also confirmed in this study. In line with previous observations from cross-sectional research (Díaz-Morales et al., 2014), the current longitudinal outcomes confirm that more family conflict is related to greater eveningness. This association was observed in both boys and girls, suggesting that family conflicts may contribute to eveningness but to a lesser extent than pubertal development. Previous studies show that longer sleep duration in adolescents is fostered by time spent with family (Sasser et al., 2021) and by more social support from parents in those experiencing discrimination (Chen et al., 2021), highlighting the benefits of communication and positive relationships with parents for sleep health. It is noteworthy that in line with these results, evening-type adolescents experience more family conflicts and more sleep problems (Ksinan Jiskrova et al., 2019); if family connectedness is related to better sleep health, evening-type adolescents may be at a greater risk of sleep problems due to both their disadvantageous sleep habits (Vollmer et al., 2017) and more frequent family conflicts.

The results of this longitudinal study are interesting because they show how girls may be experiencing more precipitating factors related to the shift to eveningness, both biological and psychosocial. The question of whether other factors are involved remains to be explored. For instance, evidence allows us to characterize evening-type adolescents as right–brain thinkers who are creative, intuitive, affective and inclined to cultural individualism and morning-type adolescents as left–brain thinkers who prefer verbal and analytic strategies in processing information and cultural collectivism (Díaz-Morales & Escribano, 2013). One may hypothesize that such individual differences may affect eveningness through a number of conflicts in the family, as thinking styles seem to be related to the resolution of interpersonal conflicts (Rafique et al., 2020).

The present study has several strengths. The age of the study sample captured an important developmental period for chronotype, which involves a developmental shift during puberty. Although other studies have considered the differences between males and females, they did not analyse a difference between boys and girls in the shift to eveningness during early adolescence. Another important aspect is that these results expand knowledge of changes in chronotype during adolescence and of the relevance of biological and psychosocial factors. Moreover, most studies that used the MESC to study morningness/eveningness are cross-sectional or measured chronotype at a single time point (Haraden et al., 2017; Haraden et al., 2019), whereas in this study chronotype was measured twice and showed an ability of the questionnaire to examine changes in chronotype across the developmental window of adolescence, so it can be used in future research aiming to capture development in chronotype over time.

This study also has some limitations. First, pubertal development was assessed by self-reports what may be less valid than objective assessments performed by a physician. The latter method, however, would be difficult to adopt in large-scale studies and is likely to increase drop-out rates among adolescents due to a possible reluctance to participate in such a procedure in adolescence. Another limitation stems from the self-reported nature of morningness/eveningness assessment. Studies using wrist actigraphy could provide more objective measures of habitual sleep parameters but require longer recording times (at least a week) and costly equipment, making large-scale research less feasible (Short et al., 2012).

## Conclusion

This research makes two remarkable contributions. First, the developmental shift towards eveningness appeared in girls but not in boys. And second, this shift was related to more advanced pubertal development and more conflicts in the family. These results have implications for shaping healthy sleep habits in adolescents and possible interventions focused on family dynamics. Sex differences in the shift towards eveningness should be considered by educators in order to evaluate the way in which this shift affects adolescents’ health and interpersonal relationships. Educational programs at school should consider that the shift to eveningness is earlier in girls, while conflicts in the family are common in both sexes. Also, the results of this study point to the importance of relationships within families for sleep, and such issues could be addressed in workshops for both adolescents and their parents. Information and advice could be provided to parents through infographics, web pages, or psychoeducation activities at high school so that they are able to understand how to deal with possible family conflicts related to the evening style of adolescents.
